# How early should be “Early Integrated Palliative Care”?

**DOI:** 10.1007/s00520-023-08213-4

**Published:** 2023-12-19

**Authors:** Cosimo Chelazzi, Carla Ida Ripamonti

**Affiliations:** Department of Medical and Surgical Specialties, Radiological Sciences, and Public Health, Università degli Studi di Brescia, Brescia, Italy

**Keywords:** Palliative care, Supportive care, Rebranding, Integration, Oncology

## Abstract

Palliative care, with its focus on comprehensive patient assessment encompassing physical, social, emotional, and spiritual pain, plays a crucial role in modern medicine. Despite its significance, integration with oncology and other healthcare specialties often occurs late in the disease trajectory. Strategies to bridge this gap include considering a “rebranding” of palliative care to “supportive care.” Early initiation of palliative care, although challenging to define precisely, aims to improve the quality of life for patients and their families. Studies show some benefits, but the evidence remains limited. An embedded model that encourages interdisciplinary collaboration between oncologists and palliative care practitioners has shown promise. However, it raises questions about training and availability of palliative care specialists. A broader approach involves integrating palliative care principles into medical and nursing education to ensure early recognition of patient needs and empathetic communication. Regular monitoring of patients’ physical and non-physical needs, along with appropriate interventions, can alleviate suffering and improve patient outcomes. Ultimately, the integration of palliative care into oncology and other disciplines focuses on addressing the individual’s needs and understanding their unique experience of suffering.

Palliative care holds a unique place among medical specialties where it has gained recognition by virtue of its approach to the cancer: treating a patient with a tumor versus the tumor of the patient [1]. Palliative care goes beyond Quality-of-Life issues common to other specialties to focus on regular assessment of a patient’s physical, social, and spiritual pain, defined as TOTAL PAIN [2] by Cecily Saunders.

Scientific research in palliative care, academic teaching and post-graduate programs are conducted in independent palliative care units and departments. Nonetheless, its integration with other health care providers and oncologists appears difficult to achieve, until a patient becomes terminally ill or enters a very advanced stage of disease [3, 4].

Strategies to facilitate integration between oncologists and palliative care teams are described below.

## “REBRANDING” palliative care

Palliative care practitioners have suggested a change of name from “palliative care” to “supportive care” to overcome the stigma which some think is associated with the former. Attempts to define supportive care and palliative care as being synonymous spurred debate [5–9] and blurred the distinctions between the two, with the risk that palliative care would lose its identity. Jean Klastersky traced the history of supportive care from initial chemotherapy for acute myeloid leukemia, where supportive care predominantly involved blood products and management of febrile neutropenia, through to the advent of cisplatin for solid tumors in which control of nausea and vomiting became a priority [10], and ultimately the control of ocular, dermatological or endocrine toxicities.

If a name creates "discomfort" for patients and physicians, how then shall we call cancer, pain, end of life, death, and dying?

## Upstream migration/earlier initiation

Some palliative care practitioners have suggested starting palliative care earlier during the cancer trajectory, i.e., defining what precisely is “early” about such care [11].

The rationale was that an earlier start would improve quality of life for patients and their families. The rebranding of palliative care could also be associated to an earlier referral, possibly because it would make the term more acceptable because no longer directly linked to end of life and dying.

Early palliative care entails empathetic communication with patients about their prognosis, symptom assessment and management, and advance care planning. Although some randomized controlled trials (RCTs) involving advanced cancer patients have reported higher quality of life scale scores and suggested a positive effect on survival in patients referred early to palliative care versus standard care, a Cochrane meta-analysis confirmed these results only with a low or a very low level of evidence [12].

In their recent meta-analysis and systematic review comparing the effects of early palliative care versus standard cancer care or on-demand palliative care on patients with incurable cancer, Huo et al. [13] reported that only 16 of 1376 studies were included. The pooled data suggested better quality of life, fewer symptoms, better mood, longer survival, and higher probability of dying at home for the early palliative care patients than for the control group. The evidence level was low, however, because of the high heterogeneity of quality-of-life measures and the few studies for the other results [13].

But what practically means “early palliative care”? Is it reasonable to ask for a palliative care consult at the diagnosis of cancer? Should “early” care be started at a particular “early” stage of disease or because patients’ needs have been assessed “earlier” during the disease trajectory?

## Embedded model (location)

The embedded model foresees interdisciplinary collaboration between oncologists and palliative care practitioners in teamwork [14–17]. This would allow space for sharing clinical information about individual patients and for integrating specialist care. For example, in their retrospective, pre-/postintervention study involving patients with thoracic malignancies, Agne et al. [15] reported that after implementation of an embedded palliative care clinic, the number of referrals for palliative care rose whereas median waiting time between referral request and first visit and time between the first oncologic visit and completion of referral decreased. Such integration may foster collaboration between oncologists and palliative care practitioners, with the added benefit for patients of shorter waiting time to first encounter with the palliative care team**.** However, what is the viability of an embedded model that connects two disciplines that differ by objectives and by training? Are there sufficient palliative care practitioners that can join with other health care professionals to provide early and integrated services for treating patients with cancer? Furthermore, how do we want to make palliative care accessible to all who need it? How do we want to reduce health inequality and mitigate unnecessary suffering? We believe that embedding alone is not the solution.

## A proposal

Our proposal is to focus on basic education in palliative care for all students during their medical/nursing education and continuous professional education for those involved in the routine care of patients with life-limiting disease or progressive chronic conditions [18, 19].

The first step is to disseminate the screening for the need of palliative care, followed by regular monitoring of a patient’s physical symptoms, emotional, social, spiritual needs, and financial distress. This can be done using simple, validated tools self-reported by the patient. Patient-reported outcomes can then help to refine and adjust interventions to the patterns of suffering, including changes in therapeutic prescription or provision of spiritual, social, emotional, or financial support.

Second, empathic communication is paramount as much as pharmacological and non-pharmacological interventions implemented according to the evidence-based guidelines for tumor and related symptoms. For this reason, health care providers, whichever their field of interest, should learn soon to communicate with their patients. While restraints on time and resources are often cited as barriers to engaging in an empathetic approach, communication and assessment of suffering are an integral part of care, if not the care itself, to which health-care professionals are deontologically committed. Set within a broader medical education program, the basics of early palliative care can be learned and then extended to all patients or those with chronic or incurable disease starting from the initial encounter, if necessary. When needed, referral for consultation with a palliative care specialist and PC team may identify and address a patient’s physical and non-physical needs. Teaching early recognition of palliative care needs through validated screening tools and empathetic communication with patients and families, may both help to alleviate emotional and physical burdens granting that all needs are timely recognized and, in case, or refractory/severe suffering, properly referred to specialists.

In conclusion, we believe palliative care can be integrated with oncology or other disciplines by centering medicine around the needs of the person. This can be done through appropriate assessment and communication. Teaching how to screen and assess the suffering early during the course of disease and the importance of empathetic comunication should be done during the course of medical training, so as to spread those concepts to the broader audince possible. This may also help trainees and future doctors and nurses to better understand when is the right time to call for a specialist referral, while providing some primary palliative care. We think that this educational proposal may work better than other strategies to implement early palliative care.

Future research is necessary to evaluate the efficacy of our proposal. This would require a commitment for all doctors to approach the field of palliative care and the empathetic approach with the patient which will be an added value to their specialist clinical skills.

## Data Availability

Not applicable.
